# Vogt-Koyanagi-Harada Disease: A Case Report With Distinct Brain MRI Enhancement Patterns

**DOI:** 10.7759/cureus.10391

**Published:** 2020-09-11

**Authors:** Ahmed El Beltagi, Mohamed Abdelhady, Nour Barakat, Yaman Alkailani, Hassan Aboughalia

**Affiliations:** 1 Neuroradiology, Hamad Medical Corporation, Doha, QAT; 2 Clinical Imaging, Weill Cornell Medicine-Qatar, Doha, QAT; 3 Ophthalmology, Hamad Medical Corporation, Doha, QAT; 4 Radiology, University of Washington Medical Center, Seattle, USA

**Keywords:** vogt-koyanagi-harada disease, leptomeningeal enhancement, labyrinth enhancement, pachymeningeal enhancement

## Abstract

Vogt-Koyanagi-Harada disease is a rare autoimmune granulomatous panuveitis with significant associated morbidity. While ocular and meningeal signs are typically observed in the acute stage, the associated classic tegumentary findings are observed subsequently. This case report highlights the imaging findings associated with this entity that serves to emphasize the role of contrast-enhanced brain MRI in early disease detection, which allows for prompt treatment initiation and better disease outcome.

## Introduction

Vogt-Koyanagi-Harada disease (VKHD) is a rare autoimmune disease that targets the ocular melanocyte-rich tissues inducing granulomatous panuveitis and diffuse T-cell lymphocytic infiltration of the choroid. Additional sites of disease involvement include the inner ears, meninges, hairs, and skin [1,2]. VKHD typically manifests during the third or fourth decade of life and is more prevalent in certain ethnic groups with greater skin pigmentation, such as Native Americans, Hispanics, and Middle Easterns [3].

It is important to differentiate VKHD from other disease entities that manifest as uveitis such as sarcoidosis, tuberculosis, and primary intraocular B-cell lymphoma. Sympathetic ophthalmia and bilateral posterior scleritis due to rheumatoid disease are also part of the differential diagnosis [2]. Although the diagnosis VKHD relies heavily on ophthalmologic examination and investigations, the classic associated brain MRI findings can serve as a useful adjunct tool to confirm the diagnosis and exclude other differential diagnoses. This case report serves to familiarize the radiologists with the classic imaging findings associated with VKHD depicted on contrast-enhanced brain MRI.

## Case presentation

**Figure 1:**
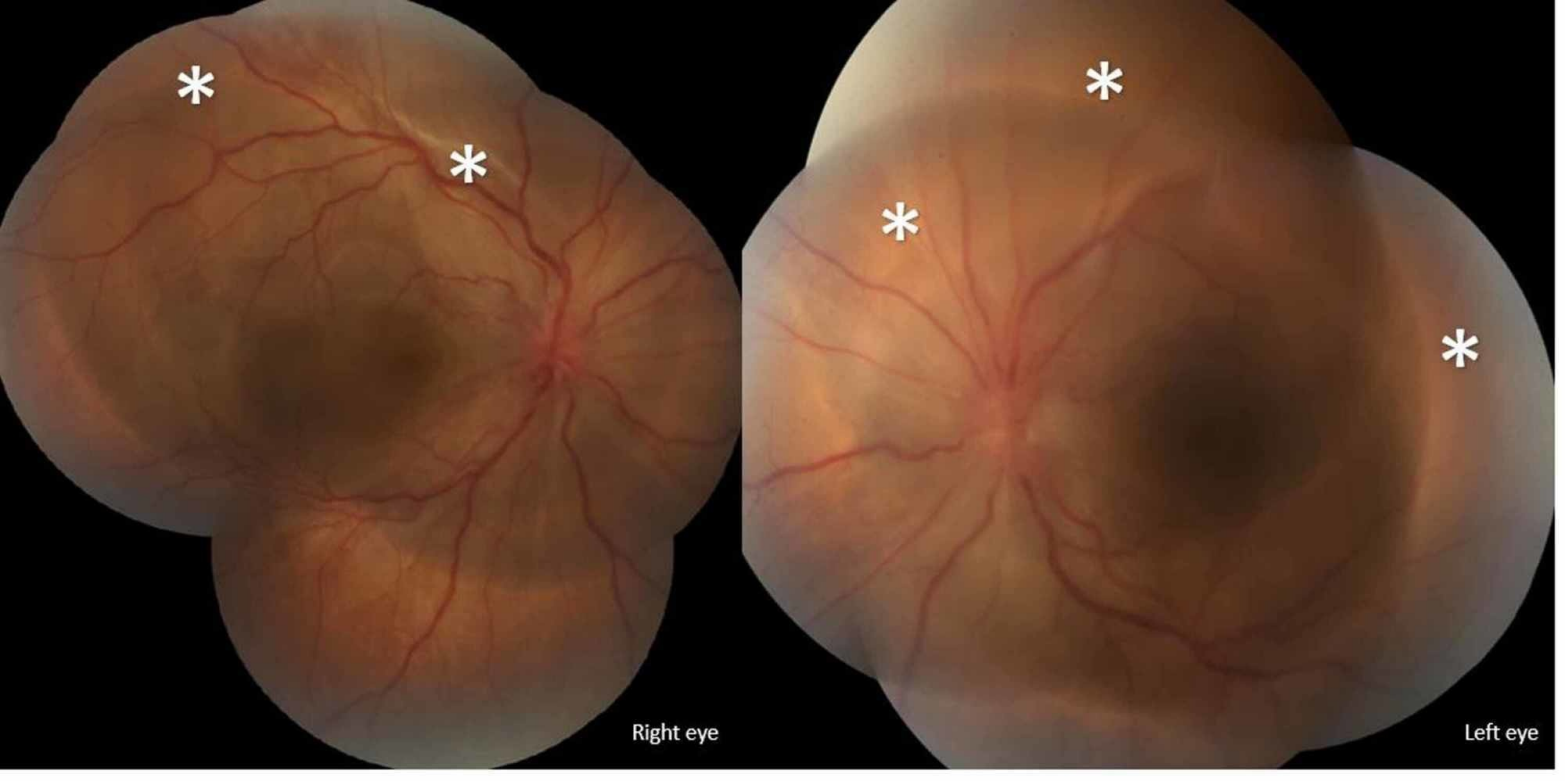
Fundoscopic examination Fundus images of the right and left eyes in the acute phase show optic disc swelling and hyperemia, choroidal whitish lesions, and multiple areas of exudative retinal detachments (asterisks).

A healthy 38-year-old male patient presented to our emergency department with low-grade fever associated with progressive headache, dizziness, blurry vision, and red eyes over the preceding week. Beside decreased visual acuity, ophthalmologic examination revealed mild diffuse conjunctival injection and bilateral sluggish pupil reaction. Further funduscopic examination displayed optic disc swelling and hyperemia with choroidal whitish lesions (Figure 1).

**Figure 2:**
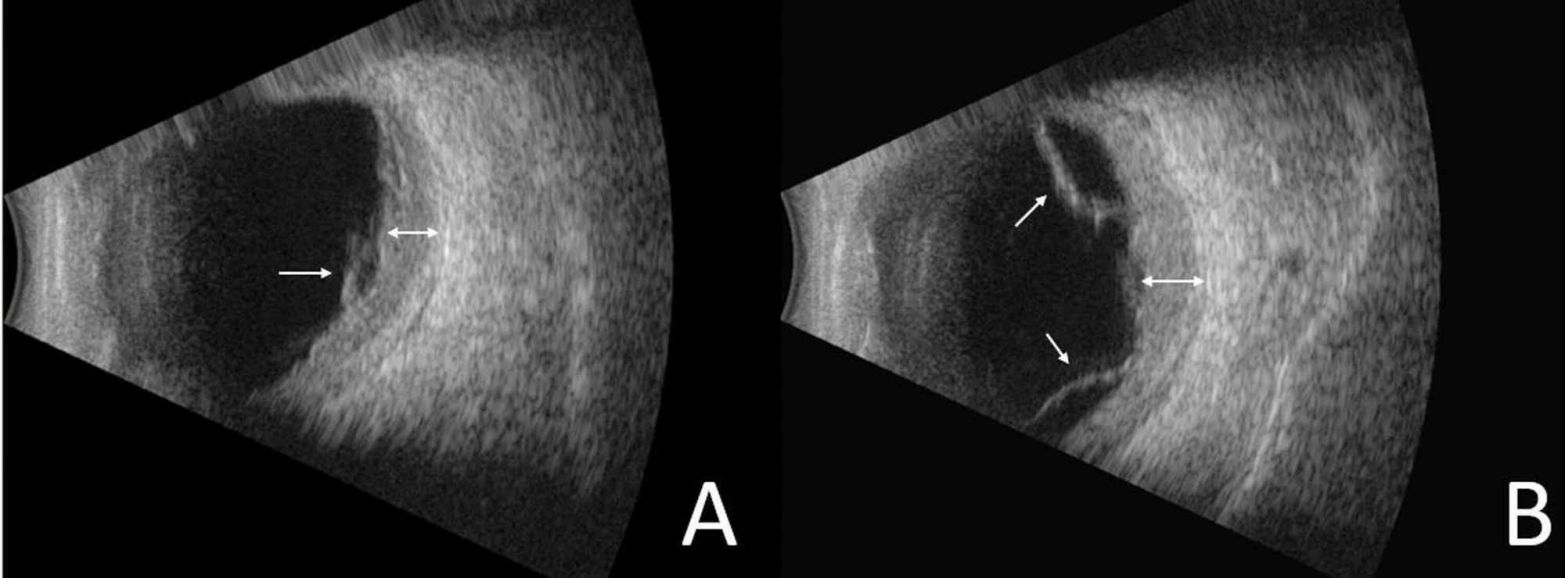
B-mode ultrasound B scan ultrasound images of the right (A) and left (B) eyes in the acute phase show bilateral choroidal thickening (double-headed arrows) and multifocal exudative retinal detachment (arrows).

**Figure 3:**
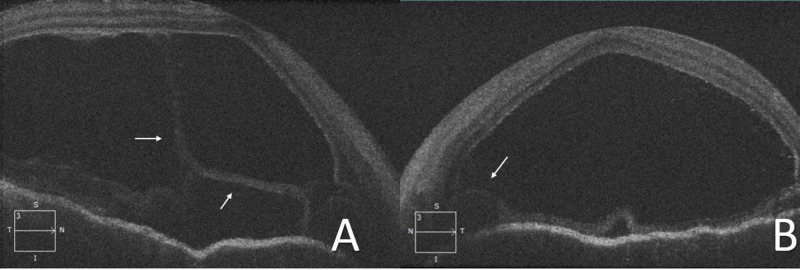
Optical coherence tomography Optical coherence tomography images of the macula of the right (A) and left (B) eyes in the acute phase show again the exudative retinal detachment and the subretinal fluid harboring inflammatory strands (arrows).

Additional ophthalmologic evaluation using B-mode ultrasound and optical coherence tomography (OCT) demonstrated significant bilateral posterior choroidal thickening and exudative retinal detachment (Figures 2, 3).

**Figure 4:**
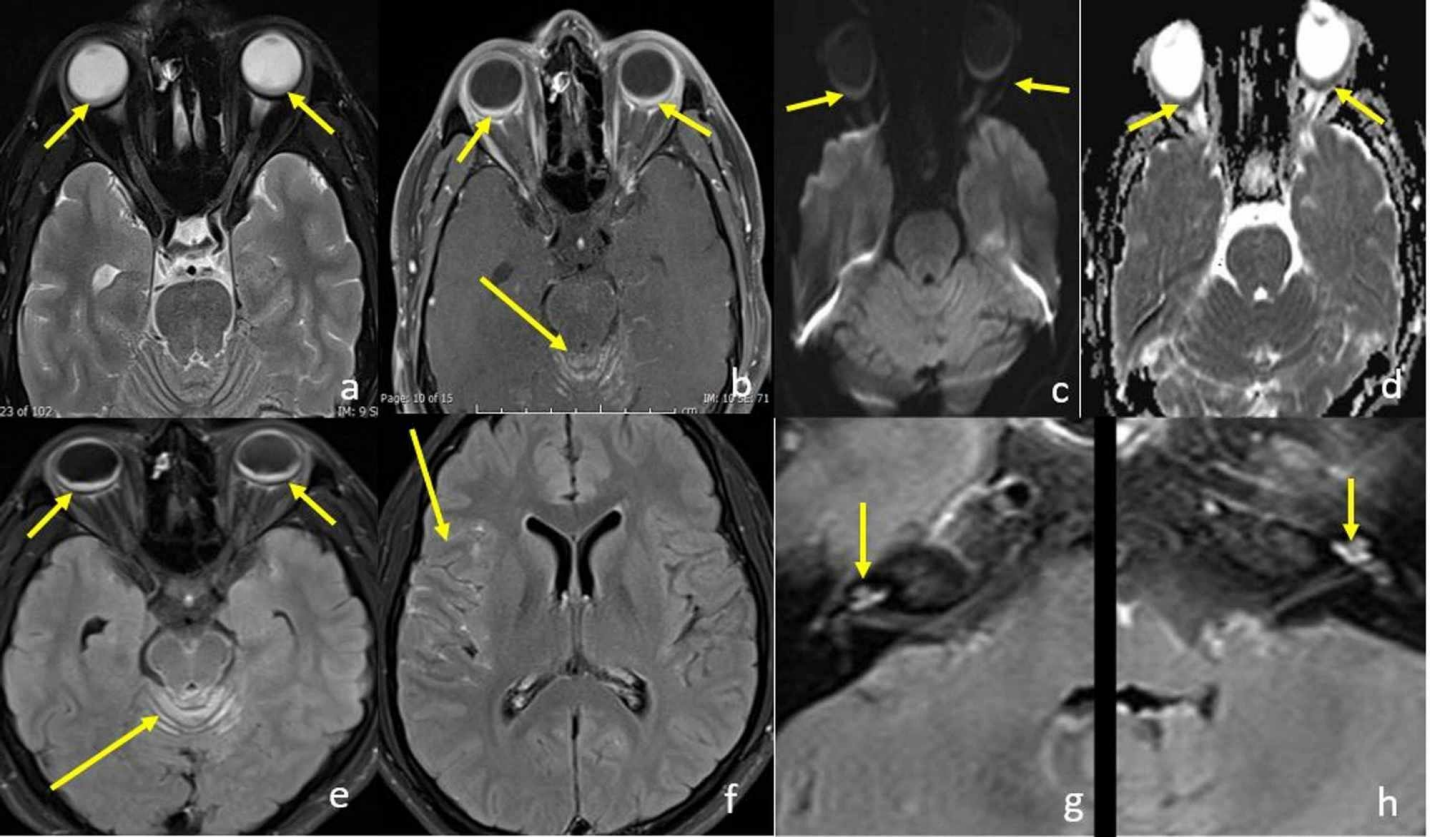
Contrast-enhanced brain MRI Axial T2-weighted MRI image of the brain (a) shows posterior predominant choroidal thickening as well as sub-tenon effusion (short arrows). Axial post-contrast fat-saturated T1-weighted MRI image (b) shows corresponding post-contrast enhancement (short arrows) as well as leptomeningeal thickening and enhancement along superior cerebellar sulci (long arrow). Axial high b-value DWI (c) and corresponding ADC map (d) show associated diffusion restriction likely related to dense lymphocytic infiltrate (short arrows). Axial post-contrast FLAIR images at the level of midbrain, (e) lateral ventricles (f), and inner ear (g and h) show leptomeningeal enhancement along the cerebellar sulci and midbrain (long arrow in e), the insula (short arrow in f), and the inner ear labyrinth (cochlea) (short arrow in g and h). ADC, apparent diffusion coefficient; DWI, diffusion weighted imaging; FLAIR, fluid-attenuated inversion recovery.

Brain MRI displayed bilateral posterior predominant nodular uveoscleral (choroid) thickening with post-contrast enhancement. In addition, there was associated episcleral thickening and enhancement inseparable from the optic nerve sheath at its globe insertion. Bilateral inner ear membranous labyrinth enhancement was noted as well. Finally, there was diffuse supra- and infratentorial leptomeningeal enhancement predominantly along the cerebellar folia and basal cisterns (Figure 4).

Finally, laboratory evaluation for infectious, granulomatous, and other autoimmune diseases was unrevealing. The patient clinical presentation and the associated constellation of ophthalmologic and radiologic findings supported the diagnosis of VKHD.

High-dose oral corticosteroid treatment was initiated, and the patient symptoms improved. The patient was symptom-free at his one-year follow-up evaluation.

## Discussion

Given the rarity of VKHD, its diagnostic criteria continue to change to reflect our evolving understanding of this entity. In 2001, the International Committee on Nomenclature proposed classifying VKHD into three categories based on the extent of extra-ocular findings: complete, incomplete, and probable [4]. Complete VKHD requires the presence of all five criteria: (1) absent history of penetrating ocular trauma or surgery, (2) no clinical or laboratory evidence suggestive of other ocular disease entities, (3) bilateral ocular involvement, (4) neurological or auditory signs, and (5) integumentary findings. On the other hand, probable VHKD is considered when only the first three criteria are fulfilled.

Ophthalmologic evaluation in the form of fundoscopy, fluorescein angiography, OCT, or ultrasound is the cornerstone to detect VHKD. However, MRI is useful not only because of its ability to detect choroidal lesion, but also can also show labyrinthine, meningeal, and brain parenchymal abnormalities. Reported brain imaging findings associated with VKHD include scattered periventricular white matter lesions as well as brain stem and peduncular lesions. Pachymeningeal enhancement is more common in the context of VKHD; however, leptomeningeal enhancement is also encountered [1,5]. In addition, MRI can show the abnormal enhancement of the membranous labyrinth bilaterally. Choroid involvement of VKHD typically affects both globes, however, near a quarter of patients starting with unilateral involvement proceeding to bilateral involvement within 15 days [6,7]. Isolated posterior pole choroidal thickening is observed in about 78.6% of cases, whereas diffuse thickening with posterior pole predominance is observed in about 21.4% of patients. Therefore, uveitis favoring the anterior pole should alert the radiologist to consider a different pathologic entity [1].

Immunosuppressive therapy for six to nine months is the mainstay of VKHD treatment to prevent disease relapse. High-dose steroids are primarily used, while other immunosuppressive and cytotoxic agents are reserved for resistant cases [2].

## Conclusions

Brain MRI is a useful adjunct tool to diagnose VKHD. Distinct enhancement patterns on brain MRI reflect the inflammatory changes affecting the choroids, cochlea, and meninges. Pachymeningeal pattern of enhancement is more common in cases of VKHD; however, leptomeningeal and labyrinthine enhancement also occurs as depicted in our case.

## References

[1] Lohman BD, Gustafson CA, McKinney AM, Sarikaya B, Silbert SC (2011). MR imaging of Vogt-Koyanagi-Harada syndrome with leptomeningeal enhancement. Am J Neuroradiol.

[2] Lavezzo MM, Sakata VM, Morita C (2016). Vogt-Koyanagi-Harada disease: review of a rare autoimmune disease targeting antigens of melanocytes. Orphanet J Rare Dis.

[3] Hedayatfar A, Khochtali S, Khairallah M, Takeuchi M, El Asrar AA, Herbort CP Jr (2019). “Revised diagnostic criteria” for Vogt-Koyanagi-Harada disease fail to improve disease management. J Curr Ophthalmol.

[4] Read RW, Holland GN, Rao NA (2001). Revised diagnostic criteria for Vogt-Koyanagi-Harada disease: report of an international committee on nomenclature. Am J Ophthalmol.

[5] Sheriff F, Narayanan NS, Huttner AJ, Baehring JM (2014). Vogt-Koyanagi-Harada syndrome: a novel case and brief review of focal neurologic presentations. Neurol Neuroimmunol Neuroinflamm.

[6] Nunes NB Jr, Bertolo G, do Amaral LLF, de Mello SMB (2013). Image findings of Vogt-Koyanagi-Harada. Arq Neuropsiquiatr.

[7] Yang P, Ren Y, Li B, Fang W, Meng Q, Kijlstra A (2007). Clinical characteristics of Vogt-Koyanagi-Harada syndrome in Chinese patients. Ophthalmology.

